# Redistribution of nitrogen to feed the people on a safer planet

**DOI:** 10.1093/pnasnexus/pgae170

**Published:** 2024-05-14

**Authors:** Helena Kahiluoto, Yousef Sakieh, Janne Kaseva, Kurt-Christian Kersebaum, Sara Minoli, James Franke, Reimund P Rötter, Christoph Müller

**Affiliations:** Sustainability Science, LUT University, 53850 Lappeenranta, Finland; Agroecology, University of Helsinki, 00014 Helsinki, Finland; Sustainability Science, LUT University, 53850 Lappeenranta, Finland; Applied Statistical Methods, Natural Resources Institute Finland, 00790 Helsinki, Finland; Tropical Plant Production and Agricultural Systems Modeling (TROPAGS), University of Göttingen, 37077 Göttingen, Germany; Ecosystem Modelling, Leibniz Centre for Agricultural Landscape Research, 15374 Müncheberg, Germany; Global Change Research Institute, Czech Academy of Sciences, 60300 Brno, Czech Republic; Climate Resilience, Potsdam Institute for Climate Impact Research (PIK), Member of the Leibniz Association, 14412, Potsdam, Germany; Department of the Geophysical Sciences, University of Chicago, Chicago, IL 60647, USA; Tropical Plant Production and Agricultural Systems Modeling (TROPAGS), University of Göttingen, 37077 Göttingen, Germany; Centre of Biodiversity and Sustainable Land Use, University of Göttingen, 37077 Göttingen, Germany; Climate Resilience, Potsdam Institute for Climate Impact Research (PIK), Member of the Leibniz Association, 14412, Potsdam, Germany

**Keywords:** food security, nitrogen, planetary boundaries, redistribution

## Abstract

Lack of nitrogen limits food production in poor countries while excessive nitrogen use in industrial countries has led to transgression of the planetary boundary. However, the potential of spatial redistribution of nitrogen input for food security when returning to the safe boundary has not been quantified in a robust manner. Using an emulator of a global gridded crop model ensemble, we found that redistribution of current nitrogen input to major cereals among countries can double production in the most food-insecure countries, while increasing global production of these crops by 12% with no notable regional loss or reducing the nitrogen input to the current production by one-third. Redistribution of the input within the boundary increased production by 6–8% compared to the current relative distribution, increasing production in the food-insecure countries by two-thirds. Our findings provide georeferenced guidelines for redistributing nitrogen use to enhance food security while safeguarding the planet.

**Significance Statement**

The divide in access to nitrogen which is critical to crop growth causes hunger, water pollution, nature loss and climate change. However, distribution optimal for food security while returning toward the planetary boundary has not been quantified. We showed that redistribution of current nitrogen use among countries could double food production in most food-insecure countries and simultaneously notably increase global production, or reduce nitrogen requirement by a third, with at most a marginal yield loss in any region. Redistribution of the magnitude of nitrogen use safe to the planet could increase food production by two-thirds in the most food-insecure countries. Nitrogen redistribution while reducing the use is possible through nutrients in residues such as manure and sewage sludge.

## Introduction

Much more inert atmospheric nitrogen has been activated for agriculture through fertilizer manufacture and intentional biological fixation than allowed by maintenance of the Earth system safe for humanity (1). The spatial divide in nitrogen surpluses and deficits leads to further transgression of the critical upper limit of anthropogenic nitrogen activation, i.e. the biogeochemical planetary boundary for nitrogen (1), while impairing nitrogen use efficiency (2). Nitrogen is a primary nutrient required in substantial quantities for cropping. Nitrogen surpluses relative to crop uptake have accelerated climate change (3) and accumulated in water systems and agricultural soils (4) of industrial countries endangering water quality and biodiversity (5, 6). Simultaneously, nutrient mining has led to a decline in soil organic matter and thus to greenhouse gas emissions and soil degradation (7) in poor countries, reducing productivity and food security (8, 9). Expansion of agricultural land to meet the food demand in regions such as sub-Saharan Africa, where the population is projected to double by 2050 (10), would cause even greater environmental problems than intensifying production through increases in nitrogen input (11, 12).

Estimates for regional environmental boundaries of nitrogen use, including recycled nitrogen, have been presented (13). Further, the impairment of nitrogen use efficiency due to the disparate spatial distribution of fertilizer use has been demonstrated (2), and the potential of agricultural management including nutrients for bridging the yield gaps has been presented (14). Moreover, various options to increase food production within the regional nitrogen boundaries, e.g. through increasing global nitrogen inputs, have been reviewed (15). Recently global yields of major cereals with two scenarios of nitrogen use were also assessed, using one process-based model only, and thus, not addressing the large model uncertainty (16). Finally, the synergetic potential of spatial redistribution of nitrogen use among countries and regions to secure food and Earth's life support system was suggested (17). However, to date, this potential has not been rigorously quantified, neither accounting for the planetary boundary and food security nor for model uncertainty. Therefore, we addressed this gap by quantifying the potential of optimal redistribution of nitrogen input for food production spatially explicit among countries and subnational regions when returning toward and to within the planetary boundary.

We estimated the potential of redistributing the activated inert atmospheric nitrogen among countries (1) to (i) increase global food production and (ii) production in food-insecure countries while (iii) reducing global activation of nitrogen. Annual nitrogen inputs through industrial fixation to synthetic fertilizers (18) and intentional biological fixation (42) were allocated to maize, rice, and wheat according to their current share of the input (14). These major cereal crops account for more than half of global cropland and fertilizer nitrogen input and 92% of the input for cereal crops (19). We employed a set of nitrogen-yield response functions from an empirically evaluated global gridded crop model (GGCM) ensemble in an optimization scheme (20–27) to assess the impact of the nitrogen input on production across the current rainfed and irrigated areas of the three major cereals, accounting for within-country patterns and allowing shifts of the fertilizer input among the crops. We report the ensemble means, as well as the range of the means for the six individual GGCMs to quantify the model uncertainty. The nitrogen input in synthetic fertilizers was redistributed among countries and crops, accounting for biological nitrogen fixation (BNF) of rice cultivation, to maximize global production with (i) the current input (190 Tg/a) (28) and with (ii) the input reduced to the current share of these crops from the planetary boundary (62 Tg/a) or the upper boundary of its uncertainty zone (82 Tg/a), reflecting the most stringent freshwater-related boundary for eutrophication (1). Furthermore, input was redistributed to (iii) minimize the input required to maintain the current global production. These scenarios were then compared to the current relative distribution of the input and to an equal distribution of current global input across cultivation areas of all countries for each crop.

## Results

### Production gains globally and in food-insecure countries

**Fig. 1.**
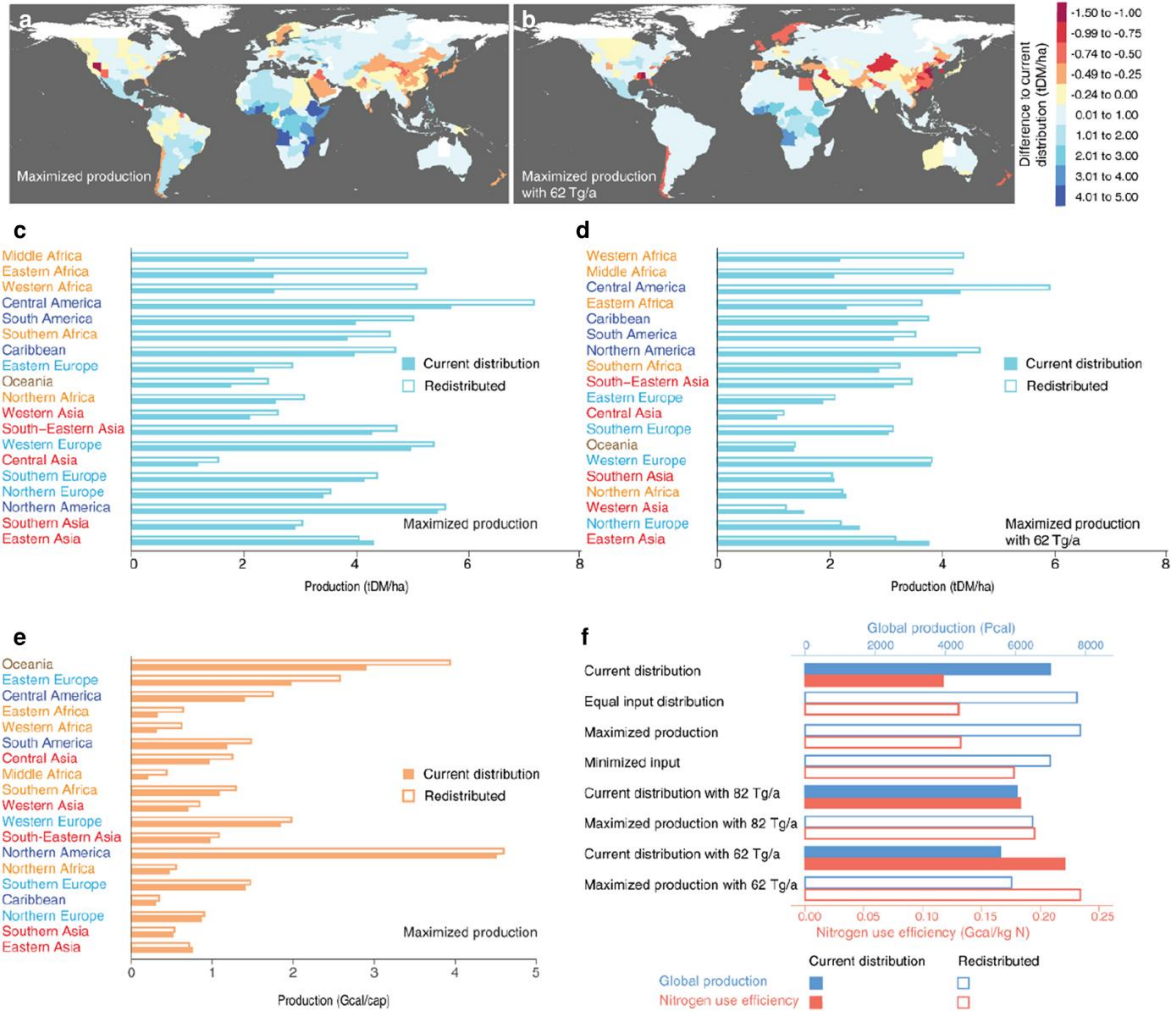
Production shifts in response to nitrogen redistribution. Differences relative to the current distribution by a, c, e) maximized production of maize, rice, and wheat with current global nitrogen input (190 Tg/a) and b, d) with the input reduced to within the planetary boundary (PB) for nitrogen (62 Tg/a). (f) Global production and nitrogen use efficiency (nitrogen input per production unit) in all the scenarios, and in the reference scenarios with current or equal distribution of the input. Standard errors of the means (SE) among the six GGCMs ranged from 692 to 755 Pcal and 0.012 to 0.028 Gcal/kg nitrogen for the scenarios. Equal input distribution represents an equal input across countries or subnational regions for each crop. Current distribution with 62 Tg/a and 82 Tg/a represents current distribution of nitrogen input reduced to within PB and the upper boundary of its uncertainty zone, respectively, i.e. an equal relative reduction of the input across countries or subnational regions for each crop. In c–e), the 19 regions (Extended Data Table S1) are in the order of a decreasing absolute gain from the redistribution, and the colors in the names of the regions differentiate continents.

**Fig. 2.**
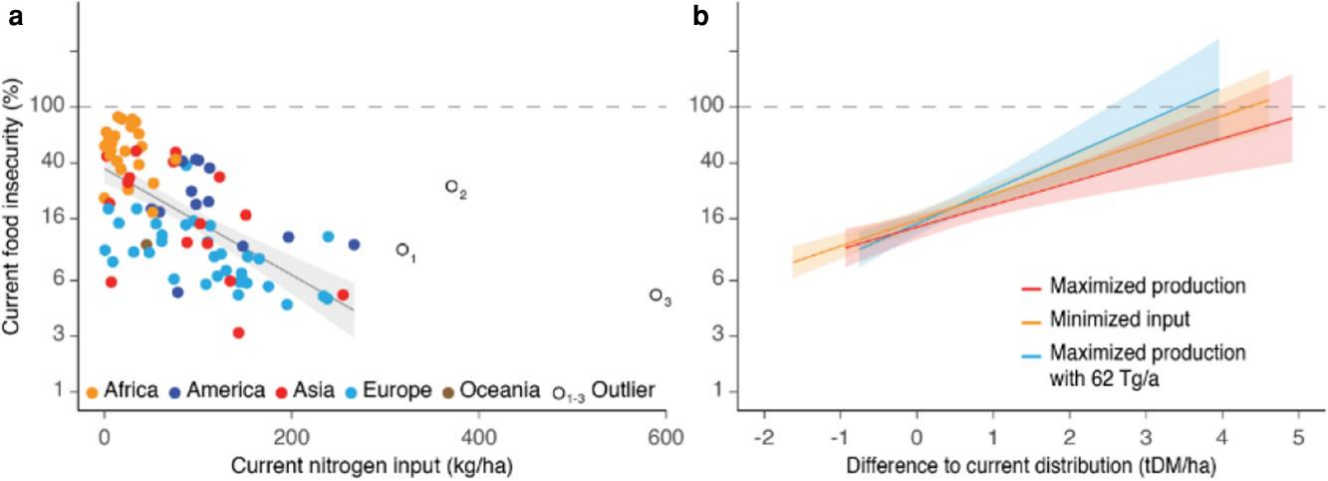
Production shifts to food-insecure countries. Pearson correlations (*r*), slopes (*β*), and 95% confidence intervals (shading) between prevalence of moderate or severe food insecurity in population (28) (logarithmic scale converted to %) and a) current distribution of nitrogen input to maize, rice and wheat (*r* = 0.642, *β* = −0.009, *n* = 88) as well as b) difference from current distribution by maximized production with current global input 190 Tg/a (*r* = 0.484, *β* = 0.423, *n* = 90), with the input reduced to within the planetary boundary for nitrogen (PB) 62 Tg/a (*r* = 0.469, *β* = 0.549, *n* = 91) and with minimized input for current production (*r* = 0.664, *β* = 0.443, *n* = 90) (all *P*-values < 0.001). For details of b), see Extended Data Fig. S3. Current distribution in PB represents an equal relative reduction of the input across countries for each crop. In a), the colors in country names differentiate continents.

**Fig. 3.**
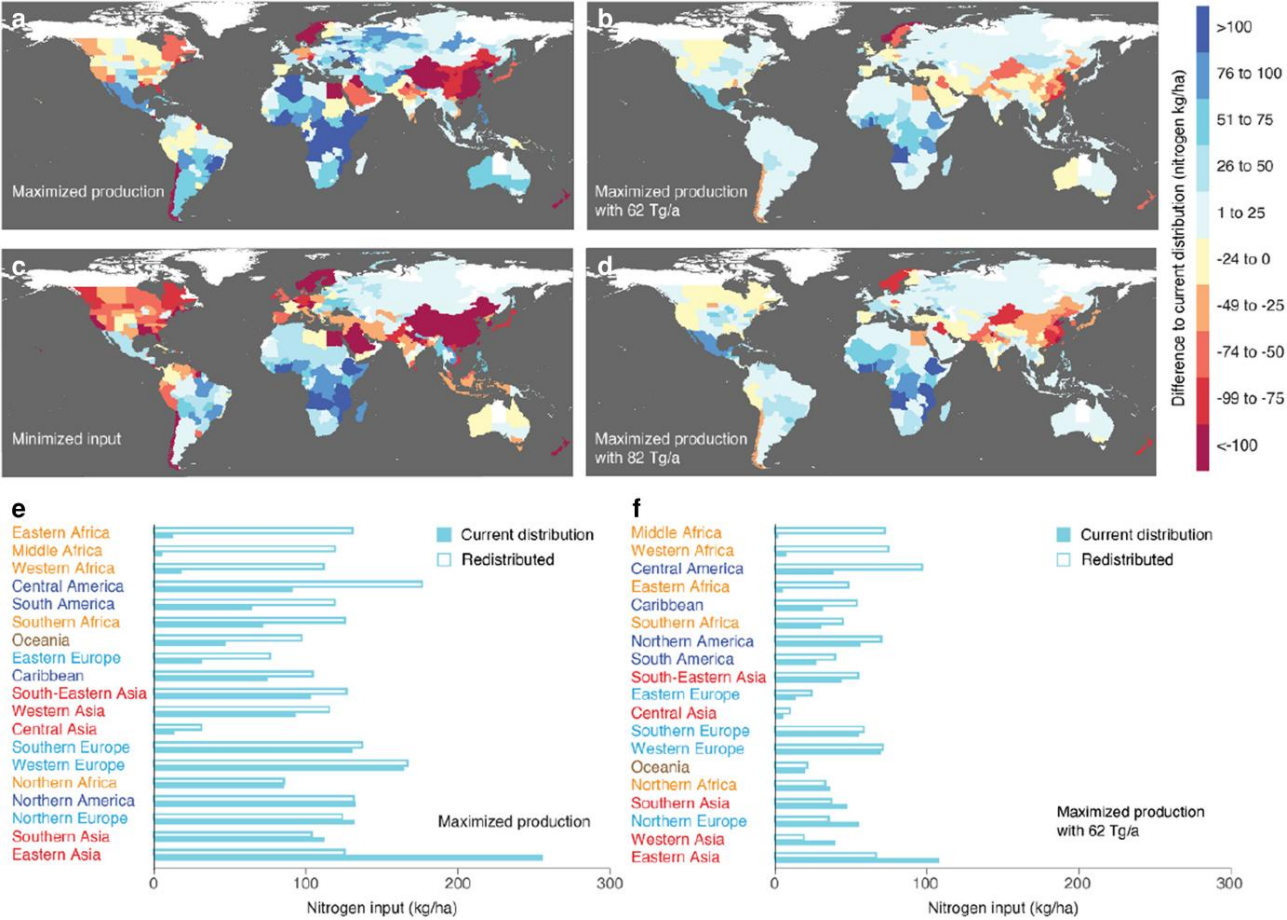
Redistributed nitrogen input. Differences relative to current distribution by a, e) maximized production of maize, rice, and wheat with current global nitrogen input (190 Tg/a) and b, f) with the input reduced to within the planetary boundary (PB) (62 Tg/a), c) with minimized input for current global production as well as d) maximized production with the input reduced to within the upper boundary of the uncertainty zone of PB (82 Tg/a). Current distribution within PB b, f) and its uncertainty zone d) represent current distribution reduced to PB and its uncertainty zone, i.e. an equal relative reduction of the input across countries or subnational regions for each crop. In e and f), the 19 regions (Table S1) are in the order of a decreasing absolute gain from the redistribution, and the colors of the names of the regions differentiate continents.

We found a 12% gain (9–19% gain depending on the GGCM) in global production upon redistribution of the current nitrogen input to maximize production (Figs. 1 and S1). Equal distribution of current global nitrogen input across cultivation areas of various countries for each crop led to a gain of 11% in production, highlighting the potential of equal access to this critical resource (Figs. 1f and S2). Because of dependence between fertilizer use and the gross national product (GDP) (29, 30), the input is currently primarily allocated to wealthy, food-secure countries (Figs. 2a and S3e). Consequently, there was a striking increase in production in countries with the greatest food insecurity while redistributing current nitrogen input to maximize global production or to minimize global nitrogen input for current production (Figs. 2b and S3a–d). For example, production in countries with moderate or severe food insecurity in more than half of the population according to the Food and Agriculture Organization (FAO) of the United Nations (28 based on household survey data on various ways of experiencing food insecurity) increased by 108–110% due to an eightfold increase in the input in those scenarios (Figs. 3 and S4). Equal input across countries for each crop increased production in these most food-insecure countries by 101% (Fig. S2). In the same scenarios, in the countries with moderate or severe food insecurity in more than half of the population, the redistribution of the input would increase production per capita by 98 to 99%, and within the planetary boundary with its uncertainty zone by 71 to 89%. These countries represent 79% of the sub-Saharan African population with the world's highest fertility rate (10).

### Production shifts among regions and countries

All continents would gain production through redistribution of nitrogen input to maximize global production. Gains in production would be greatest in Africa (more than 70%), while production in Oceania would increase by one-third and in Latin America by one-fourth. Regionally, a one-third increase in production would be achieved by Central Asia and eastern Europe also (Fig. 1c). Although there were small input reductions of 8% in southern Asia and 6% in northern Europe (Figs. 3f and S4g), these would not affect production. In eastern Asia, even though maximization of global production would reduce nitrogen input by half (Figs. 3f and S4g), production would decline only by 6% (Fig. 1c), with most of this decline occurring in China (Fig. 1a, Fig. S1a, c). However, some states in North America, India and northern Europe would also lose production, as would Iraq and Pakistan (Fig. 1a, b). Increased productivity through input redistribution was the greatest in sub-Saharan Africa (excluding South Africa), where the current production per capita, and thus food sovereignty as the control over the required food is the lowest, together with the Caribbean (Fig. 1c, e, Fig. S1). Dependence on food imports would be reduced through redistribution of the input to maximize global production in all regions except eastern Asia, which would undergo a very small loss (Fig. 1e).

### Reduced nitrogen requirement

Approximately two-thirds (53–68% depending on the GGCM) of the current nitrogen input to the three major cereals would be sufficient for the current production of those crops if input were redistributed to minimize use (Figs. 3c and S1). At a retreat to the planetary boundary with its uncertainty zone implying a drop to 33–43% of the current input, redistribution of the input would enable 84–93% (75–100% depending on the GGCM) of the current production (Figs. 1b, d and S1). Maximizing production through redistribution of the input reduced to return to within the safe boundaries would enable a gain of 6–8% in global production and of 6–7% in nitrogen use efficiency (input per production) in comparison with keeping the current relative distribution (Fig. 1f).

## Discussion

Our findings suggest that redistribution of nitrogen input has a great potential to secure food availability and sovereignty together with Earth system processes. While production gains by more equal distribution of nitrogen inputs among world's crop areas would be globally notable and the gains in most food-insecure regions substantial, the losses in production in regions with reduced nitrogen use would remain very small. Most of the country-wise decline in production would occur in China where, however, the societal benefits from reduced use of nitrogen have been shown to exceed the costs (31).

While redistribution of nitrogen input would remarkably facilitate the return to within the biogeochemical planetary boundary for nitrogen, respecting the far more challenging climate-related planetary boundary of 20 Tg/a for nitrogen (1) would also be facilitated. The climate-related boundary for nitrogen would be pushed upwards via reduced N_2_O emissions in regions of nitrogen surplus (1) and by enhanced soil carbon and nitrogen sinks in regions of nitrogen deficit (7–9). Optimization of nitrogen use within countries, in addition to among countries as presented here, would increase all benefits. Furthermore, improved agronomic management such as expanded irrigation would increase the impact of redistributed nitrogen on production, and the redistribution would enable restoration of land that has been moved out from crop production due to nutrient-depletion (14).

Other means are also needed to bridge the 7–16% food production gap remaining after the redistribution of nitrogen quantified here, relative to current global food production within the safe boundary. In addition to agronomic and technological measures the previously quantified shifts in food systems may include supply-side means (15) such as spatially redistributed cropland (12, 32), nitrogen-efficient crops (33), closing nutrient cycles through production of seaweed (34), and single-cell proteins in bioreactors (35). Dietary shifts and food waste reduction would also make critical contributions (31, 32, 36).

While food and fodder trades have enhanced food security (37) and nitrogen use efficiency (38), armed conflicts, increased export embargoes and global price volatility result in the need for food sovereignty to ensure national security (39)—and thus requires nitrogen redistribution among countries. In many food-insecure countries also the economy, and thus imports, depend on agricultural income (40) (Fig. S3f) and hence on access to nitrogen. Redistribution of nitrogen in agrifood residues and sediments that dominate water eutrophication (17) allows global reduction in nitrogen input while securing food. Technologies to capture nutrients not only in residue materials such as manure and sewage sludge but also in waste water, industrial gases, and nutrient-rich near-sediment water are rapidly developing to facilitate transportation. Our study comprised the influence of current irrigation on nitrogen response, but not that of current supply of phosphorus or other nutrients which should be separately tackled. Residues can simultaneously also provide other nutrients in deficit, micronutrients to address hidden hunger (8) and carbon for land restoration (7, 9).

Tackling current grand challenges of sustainability requires new global contracts. A comprehensive implementation of the optimal allocation of nitrogen use would be possible through regional or global regulations or economic incentives. The implementation is simplified by our finding that equal nitrogen input per crop and hectare across countries appears near-optimal. More stringent regulation and control of nitrogen pollution or use as well as of land clearing in high-input regions would help. To reduce global emissions including internal loading from sea bottom, while increasing the input in low-input countries, incentives to export residue and sediment nutrients to low-input countries are also needed. The greatest certainty of the environmental outcome would be provided by trading permissions to use or emit nitrogen with a cap defined by planetary boundary (17).

## Conclusions

Marked societal benefits in advancing food security and planetary health can be achieved through redistribution of nitrogen use among countries and regions with little loss in food production of any region, following the guidelines quantified here. Reduction in global nitrogen use is achievable simultaneously with the redistribution of current agrifood residue nutrients and past surpluses accumulated in sediments. Since the global spatial inequality in access to nitrogen is related to economy (29, 30), policies and incentives for redistribution (17) are required (41) to secure food on a safer planet.

## Materials and methods

### Overview

We derived optimal distribution patterns of annual agricultural nitrogen inputs from synthetic fertilizers (18) to maize, rice, and wheat according to the current shares (14) and intentional biological fixation (42) in rice paddy systems, across 19 subcontinental regions as well as countries and subnational regions (Table S1) under varied scenario targets. While BNF in rice systems was accounted for, only nitrogen in synthetic fertilizers was redistributed thus allowing total nitrogen input to rice of 33 to 200 kg/ha but to other crops 10 to 200 kg/ha in the simulations. To achieve the distribution of nitrogen input under the optimization targets (Data S1), we used an optimization algorithm to solve nonlinear problems after defining the initial conditions. The scenarios of (i) maximized production with current nitrogen input and with (ii) the input allowed by the planetary boundary (1) and the scenario with (iii) minimized input for current production were simulated. Planetary boundary for nitrogen of 62 Tg/a with its uncertainty zone until 82 Tg/a (1) mostly overlaps with the recent bottom-up estimate of the planetary boundary ranging from 57 to 69 Tg/a depending on the criteria (15). The scenarios (i to iii) were compared with the references of current and equal input distribution. The reference of equal distribution of the current global nitrogen input implied 119 kg/ha for maize, 160 kg/ha for rice, 100 kg/ha for winter wheat, and 75 kg/ha for spring wheat (14). The current distribution within the planetary boundary for nitrogen was represented by equal relative reduction of the input across the subnational regions for each crop within that boundary.

### Crop models

We used an emulator of empirically evaluated (20) global gridded ensemble of process-based crop models for nitrogen response in an optimization scheme; ensemble means are more robust (21) and provide better predictive skill (22) than individual ensemble members. We also quantified the model uncertainty through running the individual GGCMs and reporting the range among the models as well as SE of the GGCMs. Response functions on nitrogen-yield responses of maize, rice, winter wheat, and spring wheat were implemented in a nonlinear optimization setup, using the “cobyla” function (21) from the “nloptr” package (24) in R (25). This function represents a derivative-free optimization with nonlinear inequality and equality constraints. The response functions were derived using an ensemble of crop yield emulators, built on a large ensemble of simulations of a GGCM intercomparison (GGCMI) (26).

We included emulators of all six process-based GGCMs that contributed to the GGCMI Phase 2 data archive with simulations of nitrogen responses, i.e. the models “EPIC-TAMU”, “GEPIC”, “LPJ-GUESS”, “LPJmL”, “PEPIC”, and “pDSSAT”. The simulations served as the training domain for a set of crop yield emulators (https://zenodo.org/record/3592453#.YrAfIuxBzb0). The emulators provide 30 years’ average yields based on a set of four regressors or independent variables, such as atmospheric CO_2_ concentration, temperature, water supply, and nitrogen inputs. Current temperature and water supply conditions and a CO_2_ concentration of 400 ppm were assumed, representing actual conditions.

GGCMI emulators were built for each crop, GGCM and grid cell, but these can be aggregated in space and across GGCMs (26). Here, we aggregated emulators across the GGCMs to represent the ensemble mean response and to represent one single nitrogen response function per spatial simulation unit (national or subnational) and crop (maize, rice, spring wheat, and winter wheat). The gridded crop model ensemble was not calibrated to current productivity levels (20), and we made no attempt to do so at the aggregated level. As we compare results of the scenarios only with a simulated reference case, there is no inconsistency from that setup. With a general lack of adequate reference data for calibration (27), GGCMs are often not or only roughly calibrated and results are interpreted only in relative terms, as we do here. Harvested areas per grid cell from MIRCA2000 (43) were relied on, and these were supplemented with a map for spring and winter wheat distribution (47). The GGCMI pixel-level emulators were aggregated to the national level and averaged across GGCMs to generate one emulator per crop and country or per crop and subnational unit for larger countries. Aggregation from pixels to larger spatial units drastically reduced the computational resources required to run the optimization algorithm. The nitrogen response functions at the (sub-)national level are the result of the aggregation of gridded runs; therefore, they account for within-country spatial patterns. By aggregating to larger spatial units, some flexibility to redistribute nitrogen within countries to more productive areas was also missed. However, there is no information available on the actual distribution of nitrogen inputs within large administrative units, which are often countries, so that also the current adjustment of fertilizer inputs to spatial heterogeneity is not well represented in the reference simulation.

### Yield conversion

Calorie production across crops was used in the optimization. Crop yields in tons of dry matter (t DM) per hectare were converted to calorie production through crop-specific energy density (44) and moisture content (45). The conversion coefficient was 4.05 Gcal/t DM for maize, 3.22 Gcal/t DM for rice, 3.68 Gcal/t DM for winter wheat and 3.80 Gcal/t DM for spring wheat. Since there were only small differences between these units of production (tenths of percentages or less) irrespective of the scenario and reference, the unit is not explicit in the main text when reporting percentage shifts. Instead, shifts among countries and regions in production per capita (28) by the scenarios were also demonstrated to reflect food sovereignty.

### Statistical analyses

The relations of nitrogen input redistribution and production by the scenarios to the current prevalence of moderate or severe food insecurity (28) in the countries’ populations were analyzed using Pearson's correlation coefficient based on lm function from the “stats’ package in R (25). Additionally, the correlation of food insecurity with the current nitrogen input, GDP per capita, and the proportion of agriculture in the GDP was demonstrated to justify the analysis of the relation between the redistribution of nitrogen input and food insecurity, as well as to underpin the discussion and conclusions. Due to skewness of food insecurity and GDP, a logarithmic form was used to improve the fit. Diagnostic plots were used to assess impacts of individual countries; thus, a few (0 to 3) outliers were omitted from the analysis such as specified in the figure legends and captions. In addition to the correlation coefficients (*r*), the slopes (*β*) with their significance levels (*P*) and sample sizes (*n*) were also shown in the figures.

### Materials

We used the 31-year average yields over the historical period 1980–2010 as baseline yields. To accomplish this, we used the AgMERRA climate dataset (46), which had previously been used to train and evaluate the emulator. Atmospheric CO_2_ concentration was considered as one global value at 400 ppmv, corresponding to the concentration in 2015. We assumed a situation where nutrients other than nitrogen would not limit crop yields. Spatially explicit information regarding historical nitrogen fertilizer use was based on the GGCMI crop-specific dataset (47) (https://zenodo.org/record/5176008), which was spatially allocated (14). Current nitrogen input was represented by the average nitrogen fertilizer use for 2010–2015. In addition, BNF for rice was estimated by multiplying the rice cultivation area by the BNF coefficients (42), with the global total of 5 Tg biologically fixed nitrogen for rice by free-living cyanobacteria and the azolla–cyanobacteria association. Consequently, the current total nitrogen input to these three crops was estimated at 59 Tg/a. For the planetary boundary for nitrogen (62 Tg/a) and the upper limit of its uncertainty zone (82 Tg/a), we derived crop-specific boundaries through allocation of the global nitrogen input allowed by the boundaries to each crop according to the relative allocation for 2010–2015. The planetary boundary and the upper boundary of the uncertainty zone to these three crops were thus estimated approximately at 25 and 33 Tg/a, respectively. To aggregate yields from the pixel to the country level, we relied on cropland patterns from the MIRCA2000 dataset (43), which provide crop- and irrigation-specific harvested areas at a spatial resolution of 0.5°.

The data for moderate and severe food insecurity in the population for 2014–2016 (the earliest available data years), population for calorie consumption per capita for 2015, and GDP per capita were obtained from the FAOSTAT database for all countries available (28). Food insecurity is defined by FAO as the situation when people lack secure access to sufficient amounts of safe and nutritious food for normal growth and development and an active and healthy life. It is measured using the Food Insecurity Experience Scale (FIES), which is based on household survey data about various conditions experienced by food-insecure people. The prevalence of moderate or severe food insecurity is estimated as the percentage of people in the population living in food-insecure households. In a moderately or severely food-insecure household at least one adult has been exposed, at times during the year, to low quality diets and forced to reduce the quantity of food because of a lack of money or other resources. The probability to be food insecure is estimated using the one-parameter logistic Item Response Theory model (the Rasch model) made cross country comparable by calibrating against the FIES global reference scale, maintained by FAO.

The data for the value added of agriculture to the GDP were obtained from the World Bank database (48).

## Supplementary Material

pgae170_Supplementary_Data

## Data Availability

The data produced in the study and the R code are available via Zenodo https://doi.org/10.5281/zenodo.10965953.
