# Correction to: Redistribution of nitrogen to feed the people on a safer planet

**DOI:** 10.1093/pnasnexus/pgae206

**Published:** 2024-05-29

This is a correction to: Helena Kahiluoto, Yousef Sakieh, Janne Kaseva, Kurt-Christian Kersebaum, Sara Minoli, James Franke, Reimund P Rötter, Christoph Müller, Redistribution of nitrogen to feed the people on a safer planet, PNAS Nexus, Volume 3, Issue 5, May 2024, pgae170, https://doi.org/10.1093/pnasnexus/pgae170

In the originally published version of this article, the affiliation of authors Sara Minoli and Christoph Müller required correction and it has been updated to read as follows:

Climate Resilience, Potsdam Institute for Climate Impact Research (PIK), Member of the Leibniz Association, 14412, Potsdam, Germany.

In addition, several in-text citations were erroneous and these have been corrected as follows:

1. In the Introduction, in-text citation (19) in the following sentence has been corrected to (42):

Annual nitrogen inputs through industrial fixation to synthetic fertilizers (18) and intentional biological fixation (19) were allocated to maize, rice, and wheat according to their current share of the input (14).

2. In the Materials and methods/Overview section, in-text citation (41) in the following sentence has been corrected to (15):

Planetary boundary for nitrogen of 62 Tg/a with its uncertainty zone until 82 Tg/a (1) mostly overlaps with the recent bottom-up estimate of the planetary boundary ranging from 57 to 69 Tg/a depending on the criteria (41).

3. In the Materials and methods/Crop models section, in-text citation (45) in the following sentence has been corrected to (47):

Harvested areas per grid cell from MIRCA2000 (43) were relied on, and these were supplemented with a map for spring and winter wheat distribution (45).

4. In the Materials and methods/Statistical analyses section, in-text citation (26) in the following sentence has been corrected to (28):

The relations of nitrogen input redistribution and production by the scenarios to the current prevalence of moderate or severe food insecurity (26) in the countries’ populations were analyzed using Pearson’s correlation coefficient based on lm function from the “stats’ package in R (25).

5. In the Materials and methods/Materials section, in-text citation (18) in the following sentence has been corrected to read (42):

In addition, BNF for rice was estimated by multiplying the rice cultivation area by the BNF coefficients (18), with the global total of 5 Tg biologically fixed nitrogen for rice by free-living cyanobacteria and the azolla–cyanobacteria association.

