# Supplementary material for: Redistribution of nitrogen to feed the people on a safer planet
Source: PNAS Nexus. 2024 May 14;3(5):pgae170. doi: 10.1093/pnasnexus/pgae170 (PMC11093128; doi:10.1093/pnasnexus/pgae170)
Supplement: pgae170_Supplementary_Data [file pgae170_supplementary_data.zip › PNASNEXUS-PNASNEXUS-2023-01037-TR-s03.pdf]

Supporting Information for
Redistribution of nitrogen to feed the people on a safer planet

Helena Kahiluoto[1,2]*, Yousef Sakieh[1], Janne Kaseva[3], Kurt-Christian Kersebaum[4,5,6], Sara Minoli[7], James Franke[8], Reimund P. Rötter[4,9], Christoph Müller[7]

Corresponding author: Helena Kahiluoto
Email: helena.kahiluoto@helsinki.fi

This PDF file includes:

Figures S1 to S4
Table S1
Legend for Scenario results

Other supporting materials for this manuscript include the following:

Scenario results
The data produced in the study and the R code available via Zenodo https://doi.org/10.5281/zenodo.10965953.

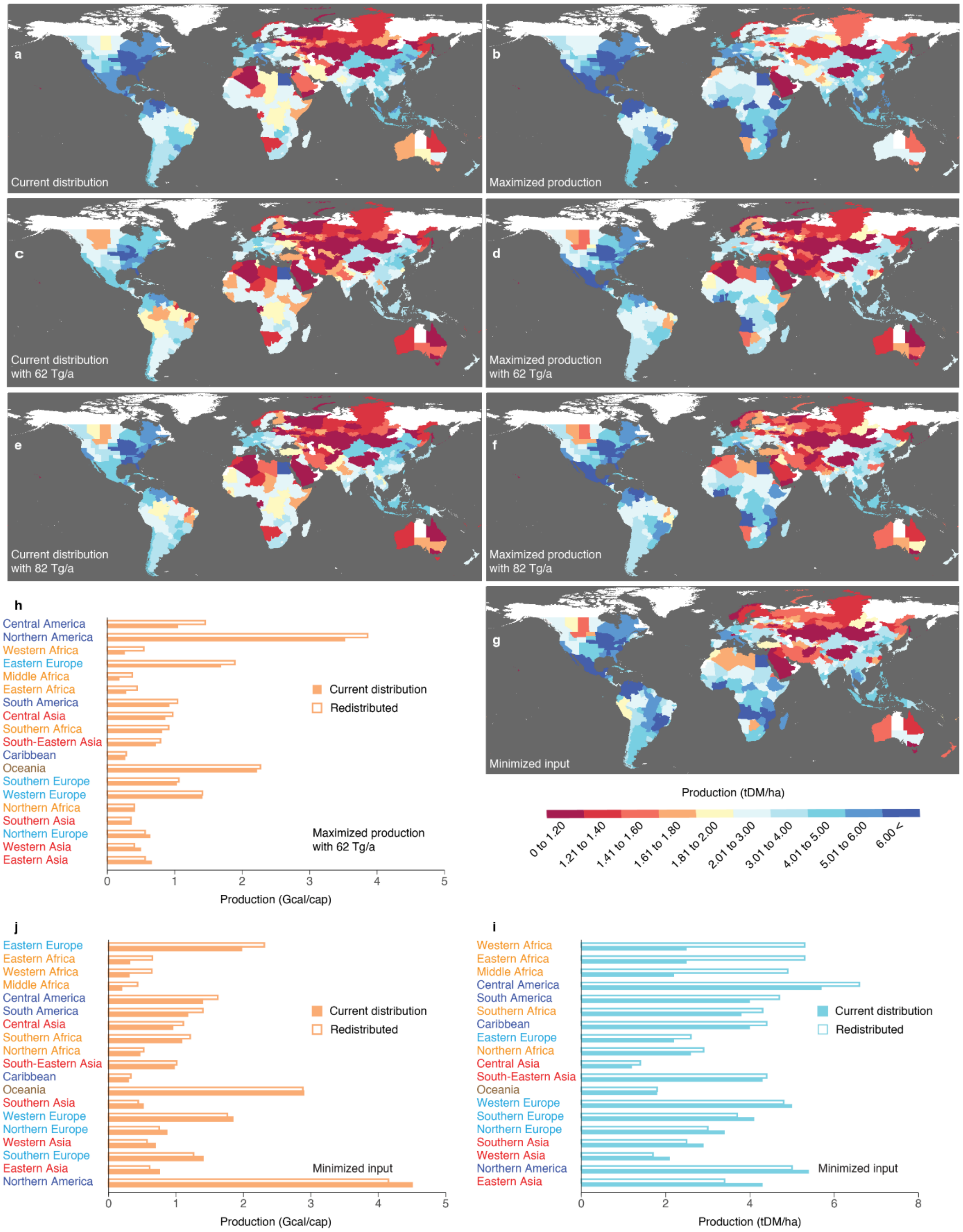

Fig. S1. Production currently and with redistributed nitrogen input. Distribution of maize, rice and wheat production (**a**) currently, (**b**) with global production maximized with current input (190 Tg/a), (**c, d, h**) with global production maximized with input reduced to within the planetary boundary (PB) 62 Tg/a, and **(e, f)** within the upper boundary of the uncertainty zone of PB (82 Tg/a), as well as **(g, i, j**) with input minimized for current production. Current distribution with 62 Tg/a and 82 Tg/a represents current distribution of nitrogen input reduced to within PB and the upper boundary of its uncertainty zone, respectively, i.e., an equal relative reduction of the input across countries or sub-national regions for each crop. In h, i and j, the 19 regions (Data Table S1) are in the order of a decreasing absolute gain from the redistribution, and the colors of the names of the regions differentiate continents.

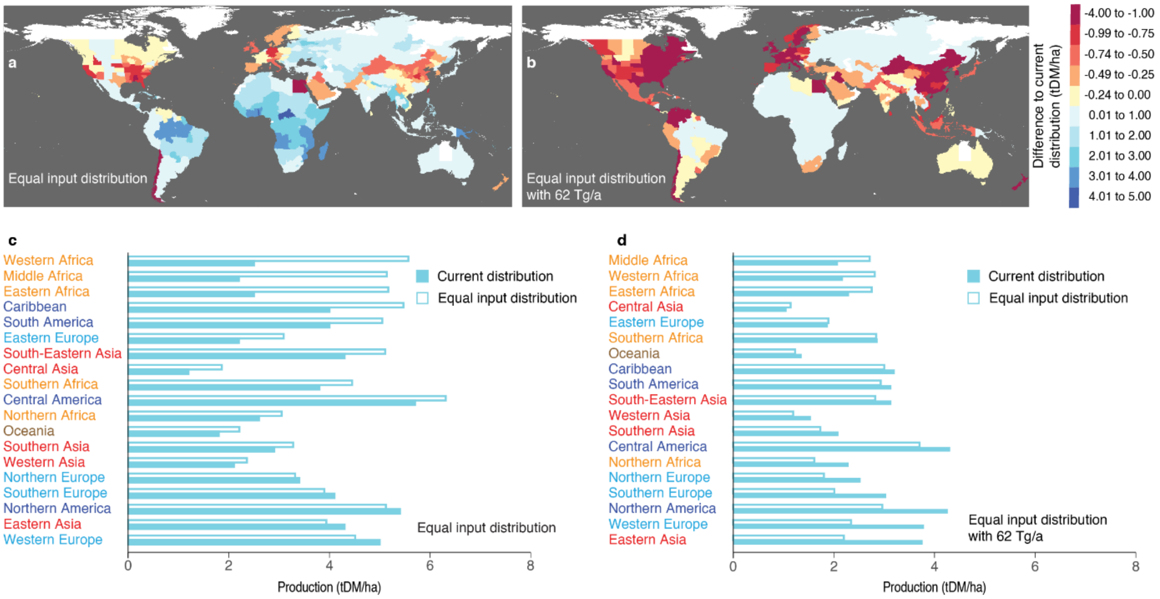

Fig. S2. Production shifts in response to equal nitrogen input across countries for each crop. Differences between the equal and current distribution of the input among countries' maize, rice, and wheat areas for (**a, c)** the current global nitrogen input (190 Tg/a) and (**b, d**) with the input reduced to within the planetary boundary (PB) for nitrogen (62 Tg/a). Current distribution with 62 Tg/a represents current distribution of nitrogen input reduced to within PB, i.e., an equal relative reduction of the input across countries for each crop. In c and d, the 19 regions (Data Table S1) are in the order of a decreasing absolute gain from the redistribution, and the colors of the names of the regions differentiate continents.

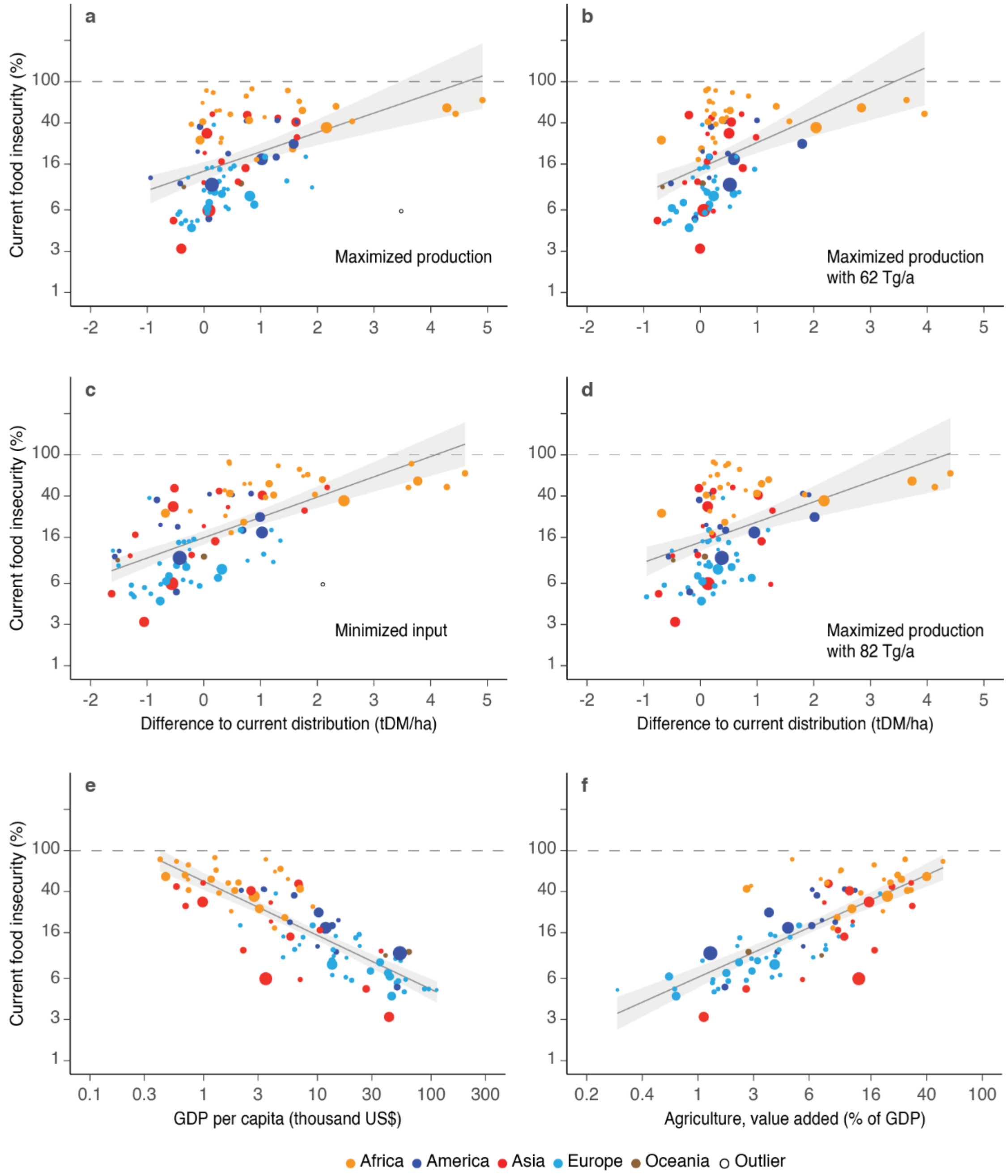

Fig. S3. Production shifts to food-insecure countries. Pearson's correlations (r), slopes (β) and their 95% confidence intervals (shading) between prevalence of moderate or severe food insecurity in the population (logarithmic scale converted to %) and difference from the current distribution of nitrogen input to the major cereals maize, rice, and wheat by maximized global production with (a) current input of 190 Tg/a and (b) with input reduced to within the planetary boundary (PB) of 62 Tg/a, (c) minimized for current global production and (d) below the upper boundary of the uncertainty zone of PB (82 Tg/a) (r = 0.440, β = 0.440, n = 90-91). (e) Food insecurity related to gross domestic production (GDP) per capita (r = 0.825, β = -0.514, n = 91) and (f) to the value added of agriculture to the GDP) (r = 0.764, β = 0.605, n = 88) (all p-values < 0.001). Circle sizes reflect the sizes of the populations of the countries; colors differentiate continents. Current distribution of the input in b and d represents an equal relative reduction of the input across countries for each crop.

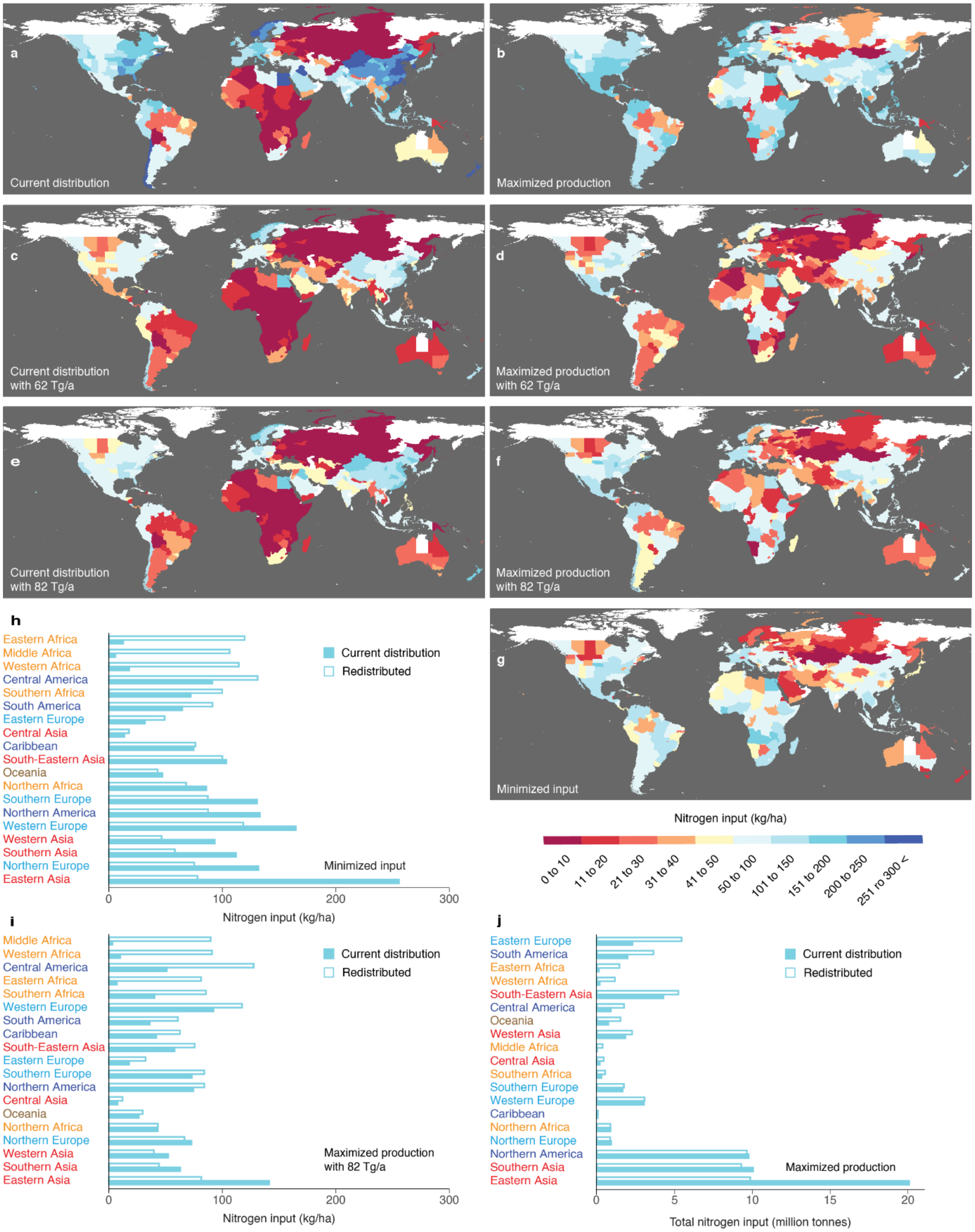

Fig. S4. Nitrogen input currently and redistributed. (**a**) Distribution currently and (**b**) with maximized global production, (**c**) currently and (**d**) with production maximized within the planetary boundary (PB) of 62 Tg/a and (**e, f, i**) within the upper boundary of the uncertainty zone of PB (82 Tg/a), (**g, h**) with minimized global input for current production as well as **(j**) for total input to maximize production. Current distribution within (c) PB and (e) the uncertainty zone represents current distribution of nitrogen input reduced to within PB and the upper boundary of its uncertainty zone, respectively, i.e., an equal relative reduction of the input across countries. In g, h and i, the 19 regions (Data Table S1) are in the order of decreasing absolute gain from the redistribution; colors of the names of the regions differentiate continents.

Table S1. Countries per region and continent. The countries included in each region and continent in Figs. 1 to 3 and in Figs. S1 to S4 (27).

Continent	Region	Country
Africa	Eastern Africa	Burundi, Comoros, Eritrea, Ethiopia, Kenya, Madagascar, Malawi, Mozambique, Rwanda, Somalia, South Sudan, Tanzania, Uganda, Zambia, Zimbabwe
	Middle Africa	Angola, Cameroon, Central African Republic, Chad, Democratic Republic of the Congo, Gabon, Republic of Congo
	Northern Africa	Algeria, Egypt, Libya, Morocco, Sudan, Tunisia
	Southern Africa	Botswana, Lesotho, Namibia, South Africa, Swaziland
	Western Africa	Benin, Burkina Faso, Cote d'Ivoire, Gambia, Ghana, Guinea, Guinea-Bissau, Liberia, Mali, Mauritania, Niger, Nigeria, Senegal, Sierra Leone, Togo
America	Caribbean	Cuba, Dominican Republic, Haiti, Jamaica, Trinidad and Tobago
	Central America	Belize, Costa Rica, El Salvador, Guatemala, Honduras, Mexico, Nicaragua, Panama
	Northern America	Canada, United States
	South America	Argentina, Bolivia, Brazil, Chile, Colombia, Ecuador, French Guiana, Guyana, Paraguay, Peru, Suriname, Uruguay, Venezuela
Asia	Central Asia	Kazakhstan, Kyrgyzstan, Tajikistan, Turkmenistan, Uzbekistan
	Eastern Asia	China, Japan, Mongolia, North Korea, South Korea, Taiwan
	South-Eastern Asia	Cambodia, East Timor, Indonesia, Laos, Malaysia, Myanmar, Philippines, Thailand, Vietnam
	Southern Asia	Afghanistan, Bangladesh, Bhutan, India, Iran, Nepal, Pakistan, Sri Lanka
	Western Asia	Armenia, Azerbaijan, Georgia, Iraq, Israel, Jordan, Lebanon, Palestine, Saudi Arabia, Syria, Turkey, Yemen
Europe	Eastern Europe	Belarus, Bulgaria, Czech Republic, Hungary, Moldova, Montenegro, Poland, Romania, Russia, Serbia, Slovakia, Ukraine
	Northern Europe	Denmark, Estonia, Finland, Ireland, Latvia, Lithuania, Norway, Sweden, United Kingdom
	Southern Europe	Albania, Bosnia and Herzegovina, Croatia, Cyprus, Greece, Italy, Kosovo, Macedonia, Portugal, Slovenia, Spain
	Western Europe	Austria, Belgium, France, Germany, Luxembourg, Netherlands, Switzerland
Oceania	Oceania	Australia, Fiji, New Zealand, Papua New Guinea, Vanuatu

Scenario results (separate file). Output data of optimization and simulation. The data structured according to the four scenarios with optimization (**A**) Maximized production (with current nitrogen input 190 Tg/a), (**B**) Minimized input (with current production), (**C**) Maximized production with 62 Tg/a (with the input reduced to within the planetary boundary (PB)), (**D**) Maximized production with 82 Tg/a (with the input reduced to within the upper boundary of the uncertainty zone of PB) and the four references with no optimization (**E**) Current distribution, (**F**) Current distribution with 62 Tg/a (the current input with equal relative reduction per country/sub-national region for each crop), (**G**) Current distribution with 82 Tg/a (the current input with equal relative reduction per country/sub-national region for each crop) and (**H**) Equal input across countries (equal distribution of current global input across countries/sub-national regions for each crop). (**I)** Food insecurity in relation to the scenarios and references, and to gross national production (GDP). All the scenarios and references and their respective global gains in production and input use per production unit (nitrogen use efficiency) are shown in Fig. 1F.
